# Meeting of the Maine Medical Association

**Published:** 1858-09

**Authors:** 


### SELECTIONS.

### *Meeting of the Maine Medical Association.*

This Association held its sixth annual meeting, at Deering Hall, Portland; commencing Wednesday morning, June 2nd, at 10 o'clock. As the proceedings have been so generally circulated throughout the State through the medium of the secular press, we are constrained to confine ourselves to a brief compendium of its transactions, devoting the major part of our record of them, so far as the Association is concerned, to the discussion of the second day.

The first day was devoted, in a great measure, to the transaction of business; the most important element of the business being the selection of two delegates to attend the examinations of candidates for degree of Doctor in Medicine, at Brunswick; the election of officers, and the appointment of a committee, consisting of one member from each county, to report, at the next annual meeting, upon the epidemics of the State. A committee of volunteers also reported themselves, to discuss some practical subject at the meeting next year, not to occupy above thirty minutes each, in their treatises.

The Association assembled at an early hour on Thursday morning — second day's session—and the Report of the Committee to whom was referred the subject of a Medical journal, at the last meeting was called for, and read. It represented the difficulties which presented themselves in the way of a publication by the Association as a body, and referred to the *Reporter* in the following language:—"But your Committee behold with pleasure, the advent of a Journal which has risen up, almost unbidden, certainly unexpectedly, of ample size, of good quality—both in mind and matter—started by a few members of this Association, who have, without any expectation of reward, brought their physical and mental energies into service, with the hope that subscribers enough may be found in our State, among their medical brethren, to encourage them to persevere in the work so well undertaken. In view of these further facts, your Committee would cordially recommend the Medical profession throughout the State, to see that the first offspring of our Medical Mater dies not ere it hardly sees the light; but fed on that wholesome pabulum, drawn from the pockets and heads of the Fraternity, it may reach the cheerful season of youth —go on with increased vigor to the period of manhood; and, reaching a green old age, be *semper aeternum.*"

The Committee on a Diploma for the members of the Association —or rather Dr. Daveis of Portland, who had acted for the Committee —reported that the result of their labor was before the assembly.— They had procured a beautifully designed and excuted lithograph, which certifies that A. B., is a member of the Association, set forth the design and intent of the organzation, and is signed by the President and Recording Secretary of the body. Beneath the certificate is a fac simile of the seal of the Association, bearing the motto, *Natura duce, Arte adjuvante,*"—"Nature, our leader," being emblematized by the North Star, and Art, our aid," by the Mariner's Compass.— This exceedingly appropriate motto was selected by Dr Daveis, the equally felicitous device, that gentleman informs us, was the offspring of the ever fertile brain of the genial poet-doctor, Oliver Wendell Holmes of Boston. The propriety and good tast of the *text* of this certificate is unsurpassed, and is happilly aided by superb mechanical execution,

The standing committee reported in favor of Waterville, as the next place of meeting, which report was accepted, and Dr. Theo. L. Estabrook was elected Orator.

The discussion of "Ulceration of the Os Uteri,—its frequency, importance, diagnosis and treatment," was then called for, and opened by Dr. D. McRuer of Bangor. We should be glad if our prescribed limits did not forbid the use of copious notes taken of this gentlemans's argument; though no report of it which we could give would do it full justice. After brief preliminary remarks, he entered with warmth upon the consideration of the topic, assuming that ulceration of the *cervix uteri* is neither so frequent or so important as many distinguished authors and practitioners believe. In support of this position, he quoted the accepted definition of ulceration, and desired a[illegible] interested in the subject to make the proper distinction between simple abrasion and genuine ulceration. He feared that many candid and ingenuous physicians had failed to make this distinction, because they relied too much upon the statements of Dr. Henry Bennett, and others of the same school. But he could prove Dr. Bennett guilty of suppression of facts, and of tampering with the truth, in his quotations. Dr. Robert Lee and Prof. Meigs of Philadelphia, have stated that abrasions have frequently been mistaken for ulcerations, but they are entirely distinct and by no means identical, in which opinion, Gibert and other Parisian surgeons coincide. Yet Dr. Bennett has stated that all the French authors agree with the views which he has made public. We regret that we cannot give Dr. McRuer's remarks, *verbatim*, as well as those of the other gentlemen who participated in the discussion; but an unfortunate defect in the acoustic properties of the Hall where the Association met, rendered it impossible to distinguish what was said, except for those in the immediate vicinity of the speakers.

Henry Bennett, pursued the gentleman, detected 220 cases of ulceration of this organ in 300 patients whom he examined,—nearly 70 per centum. Dr. Charles West found 110 cases in 268—about 25 per centum less. Dr Miller of Kentucky, regards the ratio as nearly equal. But Dr. Robert Lee had found ulceration in less than 5 per centum, out of 300 cases which he had examined.—Dr. Robert Boyd had dissected 708 uteri, yet declared that the cervical ulceration was not present. Dr. Allen of St. Marylebone Hospital, had found in 1000 post mortem examinations, which he had made or assisted at, only 20 cases of ulceration of the os uteri, of *all descriptions*, excluding cases of ulcerated cancer of the uterus, while most of those found were of a specific character. Dr. Allen alludes to the occasional abrasions of the lips , which may have been sometimes mistaken for ulceration. In 1383 cases which came under Dr. Lieber's observation, he found 4 ulcerated cervices of an unquestionable syphilitic character, and 3 cases which he designates as miliary ulceration. Many other authorities were cited by the speaker, to show the infrequency of the disease among whom were surgeons and obstetricians from England, France, and our own country; and he might go on, he said, multiplying evidence were it necessary.

The difference between the opinions quoted and those of Dr. Bennett and his followers, arises from a difference in their respective definitions of an ulcer. He thought the application of the word ulcer by Bennett, to be an abuse of terms, and abuse of terms is abuse of things—dangerous alike to the profession and the public, and likely to lead the unwary into error. But there are other sources of error. No standard can be given which shall exactly determine whether the os uteri is or is not, of normal size. According to Bennett himself, it may differ in measurement from three lines to three inches. Nor do all perfectly healthy uteri have precisely a uniform color, and the variation which is trifling under ordinary circumstances, may be much increased by modifying circumstances. The introduction of the speculum itself, may occasion sometimes pallor and sometimes an appearance of engorgement, owing to its obstructing the circulation by pressure upon those vaginal vessels which aided in the distribution of blood to the cervix.

After alluding to several distinguished authorities who deprecate the indiscriminate use of the speculum, Dr. McRuer proceeded to speak of the *importance* of the lesion under consideration. Rokitanski says that the import of a disease is in direct ratio to the value of the organ affected. Literally speaking, how is the cervix uteri so vastly important as many believe it to be? In the function of menstruation, does the cervix participate? It is even an open quesion if the uterus itself does. Neither conception, gestation, parturition, or menstruation, requires the cervix, except as a conduit. Nay, all these phenomena had been observed more than once, after amputation of the cervix.—Several authentic accounts were here quoted to sustain this position. And the gentleman could not regard the os uteri as a central organ from which such fearful results radiate.

Some other remarks were offered regarding the identity of the uterine tissue with that of other organs; and allusion was made to Bennett's hypothesis of its greater vascularity and its possession of a higher degree of vitalization. The first was a point still in dispute, and as to the latter the speaker could not reconcile the idea of vitalization with lack of nerves. (The difficulty before alluded to, rendered it impossible to gain a satisfactory idea of the argument at this point.) Dr. McRuer closed by offering the following:

*Resolved*, That ulcerations of the cervix uteri are of rare occurrence and *seldom* require the use of the speculum or the application of caustics for their cure.

Dr. Garcelon of Lewiston, rose to a point of information. Did not the gentleman say that where ulceration actually exists, there is no other means of diagnosis? Dr. McRuer intended to say no such thing. Dr. Buxton of Warren, desired to know what he did say and believe. Dr. McR., referred him to the resolution, and made a brief explanation.

The resolution was seconded, and Dr. Wood of Portland, spoke against its adoption. He was far from believing in the rarity of ulceration, and by no means limited the use of the speculum to that feature of disease. He advocated the use of the speculum, and adduced several instances in his own practice, which had greatly embarrassed him, and which could not have been treated intelligently without the aid of that instrument. He alluded to its great value in diagnosticating differentially, between some indurations, engorgements and other pathological conditions, and determining upon their malignant or non-malignant character. Many women suffer from nausea, palpitations of the heart and other constitutional disturbances during pregnancy, but they are all accounted for by her condition. But the same difficulties may sometimes occur, and frequently do occur, when they are not pregnant, and those disturbances, not admitting of being accounted for on that ground, are charged to their being "*bilious*," and so they take a dose of blue mass and a seidlitz powder, and go on to have the same routine repeated *ad infinitum*. Here then, a fault is charged upon the liver which properly belongs elsewhere. The responsibility of all diseases of women, which the other sex do not suffer, rests upon either pregnancy or "biliousness." This is wrong.—These difficulties may arise from a functional disease of the uterus, and this is the point to which the speaker would urge attention, in consideration of the subject under discussion. Let us not confine ourselves to the single feature of ulceration—to a mere symptom—but go farther. The subject, as presented to the Association, is circumscribed by too narrow limits. Are there no other diseases which require the mediation of the speculum?

The President hoped that the question might be somewhat expanded. Dr. Garcelon objected to enlarging the limits which should confine the discussion. Let gentlemen consider the subject of ulceration of the os uteri, and that alone.

Dr. Wiggin of Auburn, regarded the *cause* of this lesion as a matter of sufficient importance to claim consideration. Dr. Snow of Winthrop, called for the reading of the orginal subject of debate, as it appears on the record.

Dr. McRuer objected to enlarging the ground of debate. He did not desire to stifle inquiry, but he thought that the field which was spread before us was ample enough. He spoke of the many circumstances and symptoms involved in, and modifying, the question, as it now appears on the record. Dr. Robert Lee had demonstrated that cauterization was not, as a rule, so useful, and had proved in many cases highly detrimental, in cases of real or supposed ulceration.

Dr. Fitch of Portland, spoke in support of confining the discussion to its original limits. He considered ulceration less frequent, and when present, of less importance, than the arguments of distinguished gentlemen would cause it to be regarded. He spoke of obstinate cases which had come under his own observation, where cauterization had been employed, which yielded readily to the *vis medicatrix naturæ:* on being let alone, rapidly recovered.

Dr. Fuller of Bath, considered ulceration a very important malady, and not the least important of its features was the frequency with which it occurred. He believed it to be of very frequent occurrence, and although he did not profess the same amount of experience which he knew other gentleman had had, yet he had himself seen ulceration occasionally. Dr. Meigs of Philadelphia, who had been quoted in this discussion, had shown him cases in his own practice, which were manifestly ulcerations of the os uteri. He believed the symptom was too liable to be overlooked, and cited a case at present under treatment, in which the symptoms pointing to uterine disease had before been overlooked; but at present, he was directing treatment to this especial difficulty with eminently favorable results.

Dr. Buxton of Warren, alluded to the stigma which some attempt to cast upon the speculum by saying it is a *fashionable* instrument.—Perhaps it is; but what of that? In years gone by, if any one had a trouble in the throat, their uvula or tonsils must be pared down, or some similar operation performed. The instruments necessary for such purposes were *fashionable* , and that method of treatment was ridden as a hobby. But will any one say that no benefit resulted to the public and to the profession from those hobbies, as some scoffer calls them? The speculum was not the less valuable because it was fashionable. He esteemed it very highly, and was greatly pleased when he first obtained one. He had before been in the habit of treating diseases of the uterus by topical applications, but had found the speculum a valuable aid.

Dr. Wood asked to be excused for speaking so much, he could not help offering his testimony in favor of the speculum. He had no hobby which he wished to ride; he had no desire simply to defend a fashionable instrument, but he wanted all the light he could obtain. He did not conceive that he possessed all the information that he required in the practice of his profession, and desired to add to what he had. The opposition now displayed to the speculum is only the analogue of that which met the introduction of the stethoscope. When the latter first came in notice, many of the most eminent practitioners heaped ridicule upon it, who now value it highly. He regarded ulceration, *per se,* as trivial and comparatively unimportant; but it is worth noticing, and is of great importance, when viewed in its relation to other symptoms. He believed that many uterine diseases which had hitherto been considered incurable, or had been overlooked, can be cured. He thought many points in uterine pathology had been neglected, and desired to be understood in this discussion, not as defending any school or mode of practice, but as a seeker after truth, and as one desiring to obtain all the light he can.

Dr. Swasey of Newfield, was not prepared to advocate the adoption or rejection of the resolution now offered. It seemed in his view to embrace the most important elements of the subject proposed for discussion at the last annual meeting. He hoped that the question might not be dismissed until it had been fairly and thoroughly discussed. In his own practice, he would say although it did not embrace a large number of patients afflicted with uterine disease, he had not often met with well defined ulcerations of the cervix. This might, of course, be owing to the fact that his patients in this specialty were less numerous than with some others.

Several gentlemen offered brief remarks on both sides of the question, but we are unable to give them.

Dr. Fitch rose to explain his position. He feared from some remarks of gentlemen, that he might be misunderstood. He did not discard the speculum, but on the contrary, was happy to give his opinion in favor of that instrument. It was the indiscriminate use of it and the caustic, which he deprecated, and he thought that if the alternative of their too frequent or too seldom employment must be taken, we ought to use them less rather than more. He himself made use both of the speculum and the uterine sound, as aids in diagnosis, and highly valued both.

Dr. Nichols of Standish, thought that an empirical value had been placed upon this instrument. He was satisfied that a proper estimate of its value could not be entertained, unless the subject was approached with soberness and decorum. Some of the gentlemen had alluded to the moral effect of the instrument on delicate minded females. He thought the use of speculum gave to the medical adviser an influence which was unapproached by anything save that acquired by the accoucheur. Let us think of these things and consider the subject in its moral as well as its pathological bearing, and without levity.

Dr. Robinson of Portland, wished that there might be a modification of the resolution of to-day effected, in order that it might embrace not only the speculum, but every other agent implicated in the topical treatment of uterine disease.

Dr. McRuer interposed an explanation. He did not repudiate the speculum, but made use of it himself, and thought it a valuable addition to the professional man's instrument case. It was not the use of the apparatus that he objected to, it was the abuse of it. He thought that gentlemen must agree with him, that its employment in the cases of young girls, or very delicate and sensitive females, was exceedingly objectionable unless it were indispensable.

Dr. Wood suggested the reference of the subject to a Committee, with instructions to modify the resolution and report at the next meeting. We are not deciding this question, he said, for the physicians of the United States or of the State of Main e; indeed, it is not for the Maine Medical Association, for the science has advanced too far for us to legislate upon it effectually. It is for ourselves, individually, as honest men, and as true physcians that we are to decide, and we owe it to ourselves and to our patients, that we do not lose any light which many be offered us.

After the discussion, the hour of adjournment having nearly arrived, on motion of Dr. Robinson, it was voted to lay the subject upon the table, and renew the consideration of it at a future meeting. Adjourned to half-pasttwo.

*Afternoon.* Called to order at half-past two. As many of the members had left the city, there was no quorum present, and an informal meeting was held. The President read a letter from Prof. Peaslee of the Maine Medical School, in reply to some allegations said to have been contained in the reports of committees appointed by the Association.

Sundry votes of thanks were passed and the Association adjourned to meet at Waterville, on the first Wednesday in June, 1859.

---

THE SUPPLEMENTARY PROCEEDINGS.

Dr. Daveis' address,—of which the following is but a meagre and imperfect sketch, not, in any sense, worthy the name of a report—was delivered on the evening of the 2nd of June, at Deering Hall. It was our wish to present it in full in these pages, but we could not have done so without violating the wishes of its esteemed author, who modestly underrating its merits positively declined giving his consent to its publication. Without any intention of being offensively complimentary, we must be allowed to say that his withholding his consent to the issue of his oration is an injustice to himself, and a source of regret to the public; since the unfortunate acoustic peculiarities of the Hall where it was delivered, rendered an appreciative hearing utterly impossible. But we present our readers with the following outline of the effort, trusting that the scruples which the gentleman entertains against appearing in print many be overcome in future.

The orator commenced by renewing to the President and members of the State Association the welcome which it had been his privilege to extend to them in the morning. He hoped that the re-union might prove indeed profitable to them, and that the relaxation from physical and mental toil, which they gained during their stay in Portland and on their journeys, might refresh them for renewed efforts, on their return to their respective fields of labor.

The importance of action is evident in every undertaking which associated designs the elevation or improvement of humanity. At the present time, whichhas been properly called the age of enlightened Christian philanthropy, this power of association is especially prominent. To ourselves, as physicians, it is vitally important. Individual efforts, to whatever degree they be characterized by energy, or however numerous, must ever fail of producing the grand results which flow from concentrated action and judicious comparison. The advantage derived from concerted labor must be more permanent, and general, than that resulting from isolated endeavors, however intelligent and highly educated the individual labors may be. Beside the animating influence of emulation arising from co-operation and collective action, we lose the opportunities of improvement which might be gained by direct interchange of thought, and intercourse with those whose circumstances afford more ample means of obtaining information regarding all, or special branches of our art. We fail to secure that amount of good which we desire, in the attempt to effect it by solitary effort, regardless of the general interest of all engaged in the same calling. This, surely, is the common opinion, and this has led to the organization of the Maine Medical Association, whose anniversary we celebrate to-day.

Underlying all these facts, however, the question—To what, and to how great an extent have the science and practice of medicine been beneficial to mankind, is of vast import. Cabanis writes:—"In order to study and practise medicine in a proper manner, it is necessary to be impressed with its importance;—and to be impressed with its importance, we must believe in it." These words embody an idea which constitutes the moral base of all medical practice. The speaker here urged the necessity of confidence in the efficacy of his art, without which the physician can never properly study or practice. He quoted the reply of Luther to those who asked him if he were qualified to ask admission to the University of Erfurt. "He who prays as he ought," said the afterwards great Reformer, "has already finished half his labors and his studies." The faith which prompted that reply is the same which, in every good cause, effects its ends. And whoever aspires to dignity and success in the practice of medicine must possess a certain degree of this faith—none can ever become eminent without it. This the "*Dos pou sto*" of Archimedes. It symbolizes the faith which our Lord urged upon his disciples—that which should enable us to remove the mountains that obstruct our way; and without it, we may sink, like the doubting Apostle, beneath the waves of care that so often threaten us. Even the humblest calling is elevated by the faithful and conscientious discharge of duty; while the noblest is degraded in the individual when faith in its worth and power are wanting.

Unquestionably the confidence formerly reposed in the art of medicine has, of late, been impaired, and skepticism is rife, as to its effectiveness in curing maladies. On all sides there are those who repudiate it as a science, and refuse to acknowledge its beneficial influence as an art. This is not altogether unnatural. The reaction from blind credulity to equally blind incredulity—from faith in the Berkleian Tar Water, the royal touch, and other long exploded nostrums, to lack of confidence in simple and rational medicine—is as natural as the swing of the pendulum. Underlying all is that deep problem—what constitutes medical evidence? There is not, in my opinion, a more correct test of a physician's ability, than the care and judgment which he exercises in medical testimony. The speaker here quoted from Lord Bacon, showing that in every other art or science, its votaries were estimated by their power and ability, while the physician is judged, not by the propriety of his efforts but by their results. He thought that the public can never be a competent judge of this result.

Carlyle speaks of the present age as "above all others the Mechanical age." "It is he says, the age of Machinery in every outward and inward sense of that word; the age, which, in its whole undivided might, forwards, teaches and practises the great art of adapting means to ends," with more to the same effect. But not only is the external and physical managed by machinery, but the internal and spiritual. A state of great unrest, and a desire for the new have kept pace with civilization.

Nature, too, is imperiously questioned—but not with silence and patience, with moderation and philosophy, as by a Newton and Franklin—and when, as ancient Pythia, she is forced to ascend the tripod by some modern Alexander, the "*Aniketos ei*" is taken for the desired response and received instead of a further oracle. And so as of old, the reply which was thought to proceed from the oak, is only the voice of a human being concealed in its hollow trunk.

The age, then, is a labor-saving age—an age of change. And how gladly would we believe that the changes are for the better, and that the old which is cast aside is replaced by something which is better as well as new—that death of old opinions foreshadows the birth of more truthful ones. Still, would we gladly believe, that authors die, and their volumes moulder in forgotton dust, that the truths which they inculcated are perpetuated and cannot die. Better is it to esteem more highly the dignity of life, and believe that he has not lived in vain, who has been impelled in his labors by the desire to do good.—Believe indeed that the Unseen Hand which forms the myriads of the silicious shields of the minute infusoria composing the beautiful opal, has also preserved those truths which the united intellects of ages have produced; or as Charles Wesley has said, "God buries the workmen—but the work goes on."

I think that in this reaction from past credulity to present skepticism, lies the foundation of the doubts which are entertained regarding our science. And I would ask your attention to a few thoughts upon the *difficulties* which *inevitably attend the study of medicine as a science, and its practice as an art.* The term "medicine" is used as nearly synonymous with *disease.* By the science of medicine is understood an orderly arrangement of all the precise knowledge which we have of disease; and by its study we mean the cultivation of that knowledge with the view to increase its amount. It is intended to speak briefly of the difficulties attendant on obtaining an accurate and precise knowledge of disease.

To understand disease we must first understand health, and this knowledge we derive from Anatomy and Physiology. The former treating of *structure,* is almost entirely appreciable to sense, but great obstacles oppose a perfect knowledge of the latter, treating as it does of the *functions* of the different parts of the fame. The physiologist seeks to acquire a knowledge of these functions, by examinations with the microscope, by experiments on animals and observations of the human subject. All these branches of study are beset with difficulties. The microscope yields very different results to those more or less experienced, or who are led astray by their imagination. Experiments on animals, though highly beneficial, must be attended with difficulties, since no one of the lower animals is a perfect analogue of man. Observation on the human body present equal, perhaps greater, difficulties, for direct experiments cannot be made upon the healthy body, and disease, except of the nerves, is an inefficient instructor. What serious obstacles, then are in the way of studying healthy functions! How few are there who possess the patience, and how rare are the opportunities, to prosecute this study, embracing as it does, every age, sex, and variety of constitution. The study of certain functions of the body involves the use of instruments, and it is almost impossible to make observations on a sufficient number of persons to obtain the accurate knowledge of which we are in search. The same difficulties obtain in chemical examinations of healthy secretions. Another source of fallacy, and one which can hardly be guarded against, is that in these observations of healthy functions, those suffering with slight deviations from health, are commonly classed among the healthy.

With this necessarily limited knowledge of health, the physician commences the study of disease. These functions, with which he is so little acquainted, are disordered and changed in character, not separately merely, but in groups. He first makes an attempt to give a name to this series of phenomena that he may compare the case with the treatises in his possession, and profit by the experience of the authors.—Here we encounter another difficulty, that of writing accurate histories of disease.

The difficulty of description is always proportioned to the number of objects to be described, and in this respect disease has no superior.—The description of disease must include the *past*, *present*, and *future*. The previous habits of the patient, former diseases, and the various influences which might affect him, constitute the *past*—his existing symptoms, for many of which we must rely upon his own unsatisfactory account of himself, with those intrinsic features of disease from which diagnosis is formed, constitute the present—while the minutiæ of the history of the disease, the effects of remedies, and where necessary, the autopsy, make up what may be called the future. It is unnecessary to enlarge upon the difficulty of attaining anything like accuracy in the history of a compound fact, such as we suppose. If, then, this difficulty besets the historian of disease, no less an one op poses him who attempts to make use of the history. The accuracy of detail which makes such an history valuable, calls for unusual intrepidity in him who seeks to make use of it when perfected; and the details which have been written at so great a sacrifice of time and labor, are too often cast aside as unwieldy.

These histories, however, are what shall furnish us with accurate descriptions of disease. The descriptions compose our treatises on the practice of medicine; and give us our professional nosology. The repeated failures of those who have attempted to classify diseases, are sufficient evidence of the difficulties which they have had to encounter.

Regarding the operation of remedies, too, the physician's task is no less formidable. No one who considers the subject for a moment, can fail to appreciate the first great obstacle—our necessary ignorance of the *vis medicatrix naturæ.* There is a faculty inherent to the human frame, the faculty of spontaneously repairing the effects of violence, and of opposing the morbific influences to which the system may be exposed, and of regaining health when attacked by disease. Numerous instances where this faculty has been exerted, which will readily occur to you, indicate how far nature is capable of restoring itself, without artificial aid. But it has never been determined to what extent safety admits of our relying upon this healing power. The medical man dares not withhold those remedies which have received the sanction of his own experience or the authority of those eminent in the profession, lest he should subject himself to the imputation of trifling with the health and lives of his patients. Nevertheless, accident, the neglect of invalids or their friends in seeking medical advice, or, more than all, the "*medicine expectante*" of the ancients and of the modern French school, have thrown much light upon the influence which this conservative power exerts in curing disease. Certainly we know enough both to distrust our own judgment, and to explain the apparent success of many of the so called remedies which have been palmed upon the credulous by ignorant imposters. The speaker here alluded to the difficulty of determining between two medical agents, the claims of which were based upon equally good foundations.

But medicine is not only a difficult profession, but, in our view the most difficult. Considering the human body as merely a mass of lifeless matter, it is still of that class of matter which is most difficult of study—*the organized*, and the most complicated even of organized matter—the *animal*; and, moreover, stands at the head of animal organization. The anatomist will tell you that the most intricate and subtile machinery by which man overcomes nature, is nothing in comparison with this, and after volumes have been written upon its complexities the subject is still fresh. He will speak of the circulating system, of, its pipes and conduits, of the nervous system, with its branches and tributaries so skilfully protected from external injury; he will tell you that though he detects a centre and source of power, and a provision for its distribution through the frame, he knows nothing as to the nature of that power, nor can he perceive in the arrangement of any analogy, however remote, with the ingenious contrivances by which man extracts from matter the means of its own subjection to his will, and transmits the mandates of that will almost with the velocity of thought itself.

The microscope aids us still farther in this pursuit, even to the determining of the ultimate particles which make up the different fluids and solids, of which our frame is composed. Chemistry finishes the history begun by Anatomy. How confidently does the chemist contemplate elements which occur in his manipulations! how do invisible agents attend to do his bidding! yet when he approaches the human frame, how limited are his resources and his knowledge. He can resolve nearly all the fluids and solids of that body to their original ele_ments, but he knows not how those elements are combined, nor can he, with all his art, recompose a drop of the fluids, or an atom of the texture which he can so readily analyze.

But this organized body is endowed with life:—The blood circulates through countless tubes; the glands constantly pour out their various secretions; and the nervous system presides over each function with perfect precission. The body is still but the nervous tenement, and the nervous centres the material instrument, of a mind, which thinks and feels aud wills. This complicated union of mind and body is exposed to innumerable influences, all of which produce some effect, either favorable or otherwise, upon the frame. As Jeremy Taylor quaintly but beautifully says, "Autumn with its fruits provides disorders for us, and winter's cold turns them into sharp diseases;—and the spring brings flowers to strew our hearse, and the summer gives green turf and brambles to bind upon our graves. Calentures and surfeits, cold and agues, are the four quarters of the year and all minister to Death—and you can go no whither but you tread on dead men's bones."

It is not difficult to conceive how a frame so varied and intricate in health, should offer a study of the greatest difficulty when diseased; yet it is competent for the medical man alone to estimate these difficulties, adequately. It is because the public does not rightly understand medical evidence, that imposture overstalks the credulous.

How may these difficulties be obviated? There is no royal road to knowledge, and industry is indispensable to scientific attainment. Our own science especially demands industry of those who would distinguish themselves in it. And no profession so particularly requires that each one engaged in it should observe, think and reason independently; for the best part of a physician's knowledge is that which his personal experience and observation have taught him, and which he can impart to others in a limited degree only.

Nothing is truer than Mr. Liston's remark that "years are not the measure of experience. The greatest nnmber of well-assorted facts on a particular subject, constitutes experience—whether the facts have been culled in five years or in fifty." It is indeed so. For he whose practice embraces only ten years, may possess more of these well-assorted facts, than have been gathered by many practioners of half a century. The great requisite to intelligent practice, then, is industrious observation; a genuine union of thougnt and perception. To employ the senses, merely, is not observation, nor to exercise them frequently, experience.

If the difficulties of our profession have occupied a larger portion of this address, and the means of overcoming them a less—it is because those who listen have doubtless converted the former into a means of improvement and development, and because it is believed that these very difficulties are to the audience incentives to renewed and more vigorous efforts. It is not forgotten that those are present who, realizing the arduous nature of their calling, have ever lived and wrought in that medical faith so emphatically expressed in the newly adopted motto—"Nature our Leader—Art our Aid"—who, believing that nature may do much in her own behalf, believe also in the beneficent power of judicious Art—who believe with Chomel that the first axiom in medicine is to do no harm—and who never forget that most solemn truth, "there lies no writ of error in the grave."

Mr. Longfellow tells us in his Hyperion, that "when the learned

Thomas Diafoirus wooed the fair Angelique, he drew from his pocket a medical thesis and presented it to her as the first fruits of his genius; and, at the same time, invited her wiih her father's permission, to attend the dissection of a dead body on which he was to lecture." I have not forgotten that a part of my audience is eomposed of that gentler sex for whom such subjects as the present, can posses comparatively little interest. Yet to them I have ventured to introduce it. For I have remembered that woman—"last at the cross, and earliest at the grave" has ever fulfilled those duties by the sick bed, for which man's more rugged nature incapacitates him; and with her gentle voice and never wearying love has soothed and sustained him when human art availed not. For her, the "sweet, sad music of humanity," has ever been "that wild music, as of wind harps, floating around as in fitful spells—but soft, sometimes, and pure, and soul entrancing, as the song of angels." And when the silver cord is loosed, upon her voiceless prayer has the spirit mounted in its empyrean flight; and she who smiled first upon our cradle, weeps the longest at our grave.

Dr. Cheyne of Edinburg, is said to have acquired much of his popularity, by *little attentions*, as well as by his professional skill. And your own experience, doubtless, corroborates my opinion, that to woman, in her varied relations of mother, daughter and wife, we do, and ever shall, owe much that is essential to the sick room, the deprivation of which no medical skill can ever compensate for. And we welcome her here to-night, as our fellow-laborer, fellow-watcher and fellow sufferer, by the bed-side of the sick and dying.

Our science, with its collateral sciences, has a yet further claim on all that is holiest and most cherished in woman's heart, in this; that as science is attaining its zenith, the shadows which it was wont to cast, as its rays were falling upon Religion, are giving place to an universal light. The medical testimony which scoffers formerly adduced to overthrow the claims of Religion, have been turned upon themselves; and the world has felt that a science which teaches its professors the daily duty of going about doing good—healing the sick—causing the blind to see, the deaf to hear, and the lame to walk—is no legitimate enemy to the benevolent and beneficent Religion of the Gospel.

Chemistry and Geology, too, present the same, or similar evidence, and in the beautiful science of Botany we learn the same lesson. In an humble plant, creeping beneath his prison walls, did the atheist prisoner of Fenestrella see the irrefragable proofs of an overruling

God. And thus did the contemplative Poet of the Westmoreland Lakes, who

> "Murmured near the running brooks
> A music sweeter than their own,"

see in the meanest flower that blows—"thoughts that do often lie too deep for tears."

For all these reasons we feel that our science has a claim upon woman's sympathies, for all that is ennobling in itself, as for all that is elevating in its kindred and associated sciences. To quote a beautiful aphorism—"If we are not the Rose, we have grown up near the Rose."

With much that is cheerful in our meeting to-day, there is mingled something of solemnity and sadness in our parting. Of solemnity, because to each, how many times may the issues of life and death be committed, ere we meet again. How often will the happiness, and even the means of existence, of families depend on the faithful discharge of duty. Who is sufficient for these things? Not he alone who brings to his work the most perfect knowledge, and the results of years of patient toil, but he, too, who adds an undivided care to such amount of knowledge as he has been permitted to attain; and in the earnest discharge of every duty, relies with unshaken confidence on that Higher Power, whose fear is the beginning of wisdom. Our pleasure is mingled with sadness, also; for there is another feeling which may not be here repressed—that feeling that springs from every heart as it bids farewell to those it may not meet again. Probably not all of us may meet again this side the grave. Yet though we know not to whom the amaranth and the cypress may fall, let it be enough to know that the veil which conceals the future is woven by the hand of mercy; and that no one has lived in vain who has worked while the day lasted.

Our missions are not identical, yet do they tend to the same great end. And he best fulfils his own, whose outward and inward endeavor are one; whose faith shows through his works; whose works show that the portion of the seed field of time allotted to him has not been unemployed, but bears its fruit; and in the midst, nourished and supported by all the rest, that night flower of belief, which alone continues its bloom and refreshes with its perfume in the last darkness.

One of the most eminent of England's physicians, in speaking to his class of their duties, thus says:—"This body must be your study and continual care; your active, willing, earnest care. Nothing must make you shrink from it. In its weakness and infirmities, in the dishonor of its corruption, you must still value it, still stay by it: to mark its hunger and thirst, its sleeping and waking, its heat and cold; to hear its complaints; to register its groans." And I may add, still value it, as wisely says the good Jean Paul, for the same reason that the Turks carefully collect every scrap of paper that comes in their way—because the name of God may be written upon it.

Some may ask, whence comes the interest thus inculcated? Probably its advent is seldom natural, but is induced by a mere sense of duty. Anon the quick, interrogative principle of science enlivens and cherishes it, until it ripens to maturity. The gradations are natural—from a sympathy with the afflicted to a humane desire to alleviate suffering—and when Religion animates the heart, the dispensation of merciful deeds to his fellow creatures, becomes at once the duty and the happiness of the physician.

This is his reward from within. The reward that even the cup of cold water, given in the right spirit, shall not fail of—and that may well be theirs, whose daily round of labor is crowded with beneficence; whose nightly rest is broken that others may have better.

> " The poorest poor
> Long for some moments in a weary life,
> When they can know and feel that they have been
> Themselves the fathers, and the dealers out
> Of some small blessings."

It was this inborn yearning that gave all its beauty to the pathetic appeal of Jeanie Dean to Queen Caroline; and the recognition of which could make even the infidel Voltaire exclaim in his closing years —"I have done a little good—*that* is my best work."

In its fullest measure may this be your reward—the mercy that is indeed "twice bless'd—that blesseth him that gives and him that takes."

It has been said that this is his reward from within; yet a reward from without shall not fail him. A hearty welcome ever awaits him. The eye of the invalid brightens, and the sinking heart of affection seeks comfort in his looks. How often does he open the door upon pain, anxiety and despair, and leave behind nothing but gladness.

Even in the house of death, his presence affords melancholy consolation. Gratitude and affection attend his departing steps and his remembrance is ever sweet in the bosom of his patient.

Mr. President, and gentlemen of the Association:—My task is finished. The occasion has not been understood to require the discussion of any intricate problem or abstruse topic. Nor should I have been forward to undertake a work that might more fittingly devolved upon some one of those—older and abler than myself—whom I have had the honor to address. I have come, responsive to your call, to recognize and mark the occasion; to present some topic for our united reflection, this evening; something to aid us in the path of duty; something to sustain us in the hour of trial. With no pretence to logical precision—nor yet entirely devoid of method—but rather like that famous picture, which Homer has inscribed upon his hero's shield;—"Of things, and movements heavenly, that appertain unto a higher sphere," hemmed round and embellished by the representation of earthlier and homelier pursuits.

Happy he, who can, in some degree, realize in his daily life his ideal of professional excellence. Thrice happy, who writing his name by his daily deeds as one who loves his fellow-men, shall, like the good Ben Adhem, find at the last, that he has most effectually written it as one who has loved and served his Maker.

Sufficient for me, my labor done, if a single thought suggested finds a response in your bosoms; one that can aid, in any manner, to bear faithfully what of life and labor yet remains. To one at least—to myself—the subject has proved no ungrateful theme. It has served to impress yet more strongly on my own mind, the importance and dignity of our profession; and that all knowledge, all science, has only reached its culminating point, and answered its highest end, when it lays its offering at the altar of the Most High. That thus has religion knit together all the wants and hopes of mankind into faith, and wove an immortal crown for humanity.

---

We cannot refrain from repeating our expressions of regret, that a compliance with the wishes of the accomplished author of this address, compelled us to mutilate the greater part of it, so that he would scarcely know his own production. We deem it a simple act of justice to him, to make this statement, though there is little fear that our poor abridgement would be mistaken for the original.—*Maine Medical & Surgical Reporter.*

